# The effects of exercise training induced calories expenditure on type 2 diabetes related cardio metabolic physiological parameters and adipocytokines

**DOI:** 10.1007/s40200-021-00808-0

**Published:** 2022-09-03

**Authors:** Mahmoud Asle Mohammadi zadeh, Saleh Afrasyabi, Zaynab Asle Mohamadi

**Affiliations:** 1 Department of Exercise Physiology, Faculty of Sport Sciences, University of Isfahan, Hezar Jerib Street, P.O. Box 81746-7344, Isfahan, Iran; 2 Department of Sports Science, Farhangian University, Bushehr, Iran; 3 Department of Physical Education and Sport Sciences, Islamic Azad University Science and Research Branch, Ahvaz, Iran

**Keywords:** Calories expenditure, Type 2 diabetes, SFRP5, Cardio metabolic parameters

## Abstract

**Background:**

Recently, many studies have examined the effects of various training on pro-inflammatory and anti-inflammatory adipocytes. The results of these studies are contradictory. Some have reported positive effects and others have reported negative effects. However, there is no research to study the effect of exercise on similar energy expenditures on adipocytes. Hence the purpose of this study was the effects exercise training induced calories expenditure on type 2 diabetes related cardio metabolic physiological parameters and adipocytokines.

**Methods:**

Sixty-eight men patients with type 2 diabetes [12 weeks] were randomized to 4 groups according to training regimens. the groups are [1] HIIT [n = 17], [2] RT[n = 17], [3] AT[n = 18], and [4] AT + RT n = 16]. For 12 weeks [4 days/week, 20–30 min/season], participants performed training sessions with 300 kcal energy expenditure. Before and after 12 weeks interventions, Anthropometric and physiological variables and Glucose, insulin, FFA, LDL, HDL, TG, TC collected and analyses. Leptin, SFRP5, LGR4 and Irisin levels in Serum were assessment by ELISA.

**Results:**

Serum irisin concentrations were significantly higher in AT [%20.4] compared to other groups. Leptin, SFRP5 and LGR4 were significantly higher in HIIT [%-21.7, %48.1 and %30.9 respectively] compared to other groups. Serum SFRP5 concentrations were significantly increased in 4 groups[P > 0.05]. However, leptin and LGR4 were significantly decreased and increased in 3 groups expect in RT group[P > 0.05]. And irisin concentrations were significantly increased in AT group only[P > 0.05]. And many variables indicated positive and negative relationship between together [P > 0.05].

**Conclusions:**

The findings of the present study showed that if exercised with energy expenditure equal to HIIT training, it has the greatest effect on improving inflammatory and anti-inflammatory indicators in type 2 diabetic patients, as well as glycemic and lipid-chemical variables.

## Introduction

Diabetes mellitus [DM] is a progressive metabolic disorder identified by severe hyperglycemia correlated with compromised, lipids, carbohydrates and protein metabolism, insufficient insulin regulation or diminished response to physiological effects of insulin production by pancreatic β-cells [1]. In patients with type 2 diabetes mellitus, the occurrence of coronary heart disease [CHD] is exceedingly high, with around 50 per cent of patients suffering from cardiovascular diseases [2]. Adipose hormone dysfunction in obesity has been associated to heart disease, diabetes and other chronic diseases [3]. Adipokines is supposed to be the best dysmetabolic biomarkers in overweight, obese and diabetic subjects; and has been recommended as potentially large in the diagnosis of the prevalence of cardiovascular and type 2 diabetes mellitus in persons with overweight and obesity [4].

The potential advantageous results of Exercise training in T2DM on glucose regulation and various medical conditions for CVD are well established [5, 6]. Depending on somewhat minimal-quality information, resistance exercise in programs with a period longer beyond 12 weeks, contrasted with aerobic exercise, seems to be successful in enhancing elevated VO2max in diabetics. Nonetheless, this form of workout’s efficacy in Adipocytokines [SFRP5, Irisin, Leptin and LGR4] and glycemic regulation [Insulin, Fasting Glucose, HbA1c and HOMA-IR] and lipidemic factors [HDL, LDL, TC^1^ & TG^2^] remains conflicting [7].

Leptin has been one of the main hormones responsible for regulating the balance of energy and body mass by adjusting energy consumption and fuel costs [8]. Focused on the systematic findings of clinician-reviewed studies that regular workout induces a slight yet persistent reduction in circulating leptin rates independent of age and sex [8]. Racil et al. [2016] indicate that HIIT opposed to moderate intensity can have more favorable impact on leptin [9]. Therefore, it can be noted that exercise intensity than exercise duration has an important effect on leptin changes [10]. It is noted that leptin is an obesity indicator which improves monocyte generation of TNF andIL-6 and also facilitates the formation of ROS^3^ and induces monocyte cell proliferation and migratory actions [11]. Also revealed that Leptin by triggering the JAK2/STAT3 signaling pathway, promotes the generation of CC-chemokine ligands on macrophages [12].

Secreted frizzled-related protein-5 [Sfrp5] has been shown to be an innovative adipose tissue-released hormone which connects overweight to diabetes and is currently known as an anti-inflammatory adipokine [13]. Researches also shown that Sfrp5 provides a new goal in glucose homeostasis to regulate obesity-connected disorders [14]. As to its relationship with type 2 diabetes [T2D] in humans, reports are contradictory, but two experiments recorded lower systemic SFRP5 compared with controls in participants with prediabetes and type 2 diabetic patients [15]. Indeed, SFRP5 expresses itself strongly in WAT relative to other tissues and Also when normoglycemic, insufficient SFRP5 demonstrate insulin resistance and a fatty liver [16]. Such findings indicate that SFRP5 is a desirable goal for reduction of inflammation of the fat tissue and metabolic disease exacerbated by obesity and T2D [17].

Irisin production from Subcutaneous white adipose tissue[scWAT] and visceral white adipose tissue [vWAT] was stimulated by quick-term cycles of aerobic exercise activity, while it was reduced by long-term exercise activity and starvation [18]. In diabetic patients, concentrations of irisin were greater relative to lean mass participants, and the lean mass content was significantly associated with bloodstream amounts of irisin [19]. That means that WAT-secreted irisin can play an important role in the metabolic disorder and insulin resistance associated with obesity [20]. Furthermore, Kartinah et al.[2018] showed that the impact of HIIT on adipose irisin concentrations was seen to be substantially more efficient relative to CMIT^4^ in unhealthy metabolic conditions [18].

A children’s study has found a clear and beneficial relationship among concentrations of Irisin and Leptin [21]. Though another research has suggested that Leptin doesn’t influence the blood levels of Irisin [22]. Moreover, a research documented that LGR4^5^ expected a type of Leptin mutation to enhance glucose metabolism, indicating that LGR4 and Leptin could have activity on bloodstream glucose regulation and in pancreas tissue extremely represented and when consuming, influenced pancreas. Mir et al. [2020] suggested that 12 weeks of mixed HIIT and resistance improved bloodstream concentration of SFRP5 and reduced bloodstream value of glycated hemoglobin [HbA1c], body mass index [BMI], percentage body fat [PBF], insulin resistance [IR] relative to placebo group. SFRP5 was substantially harmful to WNT5A, insulin resistance, BMI, and PBF. BMI has had a strong favorable interaction with WNT5A, IR, and PBF. [23]. As mentioned above, SFRP5 is related to leptin and LGR4 and these two indicators effective of insulin resistance in diabetic patients. Therefore, it has not yet been determined how exercise with similar energy costs may affect these parameters in type 2 diabetic patients.

The findings demonstrate that irrespective of the mode of training, exercise training is an important technique for enhancing certain insulin resistance-related adipocytokines in type 2 diabetes patients. However, it has been noted that the type of exercise does not affect adipocytokines, but Zanuso et al. [2010] have found that compared with training volume, exercise intensity is a best anticipator of both the variation between the exercise training and the control population in HbA1c and VO2max [24]. In addition, when energy expenditure in different intensities of exercise were the Similar, can conclude that exercise with high intensity led to higher decreases in HbA1c, higher VO2max rises and higher insulin sensitivity rates. from the aforementioned study, we noticed that in patients with T2D, there are a few studies examined on influences exercise training induced calories expenditure on type 2 diabetes related cardio metabolic physiological parameters and adipocytokines. In our research, we established if the serum adipocytokine concentrations [Leptin, Irisin, Sfrp5, and LGR4] exhibited specific variance trends in T2DM patients, and linked to exercise intensity and during in the similar exercises training induced calories expenditure.

## Material and method

### The study population

This study was done using an experiment research methodology and was accepted by the Esfahan University Local Scientific Ethics board [IR.UI.REC.1396.062] in compliance with the standards of ethics set out in the 1983 Helsinki Declaration. The research was conducted in Abadan Recovery Centre, Khuzestan, Iran since November to January 2019. The study’s participation requirements are as obeys: getting type 2 diabetes for further least 3 years, being controlled with hypoglycemic oral medications exclusively, and a lack of physical activity. These factors became the exclusion criterion of the study: possessing retinopathy, nephropathy and extreme neuropathy, history of previous cardiovascular disorders, stroke, and restrictive musculoskeletal complications Reducing exercise training. Prior to performing the study, participants were required to fill personal records and surveys on readiness for workout [PAR-Q] and were directed to a medical checkup specialist. The sample size according to Kadam and Bhalerao’s analysis was estimated at 15.7 for growing category. Significant for sample size were p < 0.05, and for power of study were 85%, and effect size were 1.45.

**Fig. 1:**
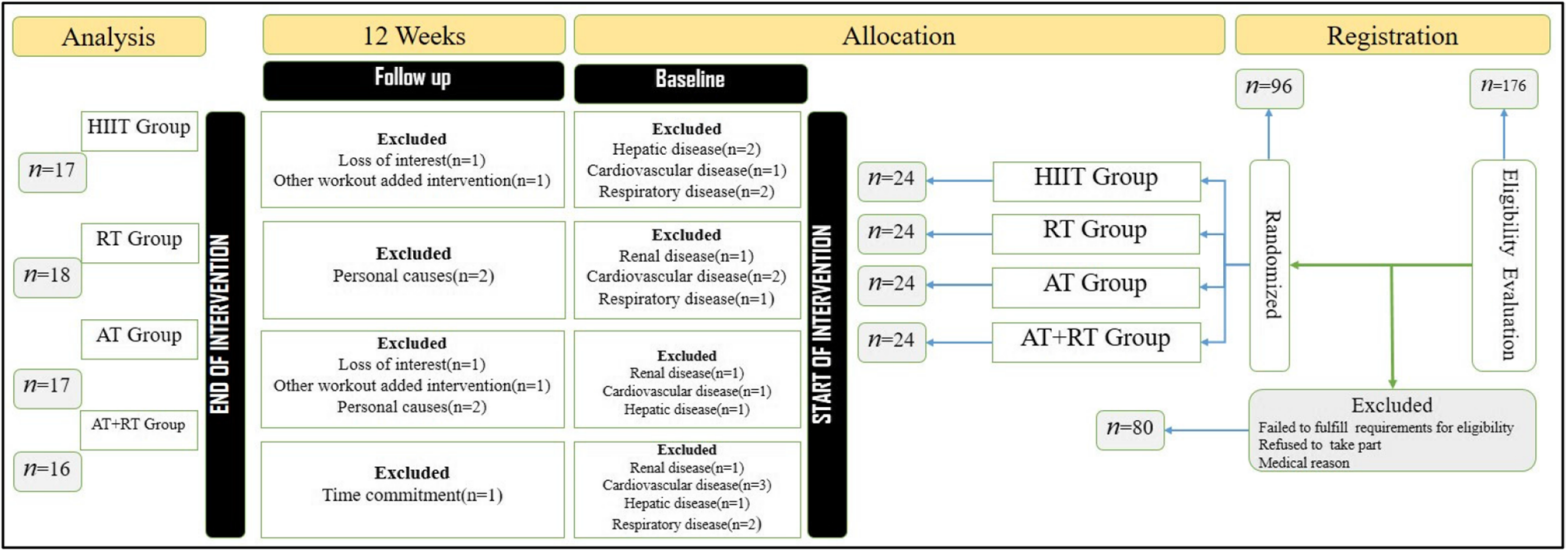
Flowchart of the study. [1] After Eligibility Evaluation, Participants randomly allocated to the Resistance Training [RT], high-intensity interval training[HIIT] Aerobic Training[AT]& RT + AT Groups; [2] Baseline assessment will be carried out immediately after evaluation and 1–3 days before start of medical care of patients; [3] Finally, participants carried out 12 weeks exercise training interventions

To confirm that the number of individuals in each category exceeded no less than 15 despite possible sample reduction, 96 participants were chosen after receiving physician consent and finally were split into four groups [24 subjects in HIIT Group, 24 subjects in RT Group, 24 subjects in AT Group and 24 subjects in AT + RT Group] utilizing the SAS program using a controlled randomization [random distribution guideline]. FINALLY, 28 participants [7 subjects in HIIT group, 6 subjects in RT group, 7 subjects in AT group and 8 subjects in AT + RT Group] were omitted from the research after the analysis due to their recording of improvements in their treatment routine and medical and exercise training interventions (Fig. 1). After providing details about the process of study, we explained its benefits and probably potential harms and then written consent was obtained from volunteers.

### Exercise program

According to the guidelines of the present study, exercise training programs were designed based on an energy expenditure of almost 300 kcal/day in 4 groups.

The HIIT program guideline composed of cycling ergometer drills using The Monark 894E Ergomedic Peak Bike[made in.

Sweden[. The participants learned to in every phase performed of 4–5-min warm-up with an interval period of 60 s [intensity: 90-100HRmax; repeat:8–12 times in 1session/day; 4 days/week] at the individual’s breaking wattage; 120 s of rest[intensity: 60–70 HRmax; repeat:9–13 times in 1session/day since first week to 12 weeks; 4 days/week] and 4 min of cool-down were followed for 12 weeks. Depending on the results and expected commitment of patients [22–32 min] who performed the 8 intervals at the first HIIT workout until 12 intervals at twelfth HIIT weeks, the wattage was changed upward by 15 percent. Indeed, for those who were unable to sustain the necessary 135 rpm at every time, the wattage was changed reduced by 10–12 percent depending on the same parameters. In fact, the wattage was changed upwards in 12 percent steps over the 12 weeks of HIIT to guarantee optimum pressure was performed for each experiment, because a participant had achieved 8–12 periods while achieving more than 135 rpm for three successive sessions (Table 1).

**Table 1:** Description of allocated exercise training induced calories expenditure procedures across categories

	HIIT	RT	AT	AT + RT
	[%HR_RESERVE_]	[%1RM]	[%HR_RESERVE_]	[%HR_RESERVE_]/ [%1RM]
Intensity [%]	90–100	55–80	70–80	70–80
Heart Rate[bm]	151–168	-	118–134	120–140
Interval	8–12	18–8	1	1 + 5–9
Duration	60[s]	15[s]/Rep	20–25[Min]	20–25[Min] + 15[s]/Rep
Recovery intensity [%]	60–70	90–150	-	-
Heart Rate[bm]	101–118	-	-	-
Recovery duration [s]	120	-	-	-
Recovery Interval	9–13	Stretch	aerobic	Stretch- aerobic
Total time commitment/day [min]	22–32	27–33	≥ 20	≥ 20 + 27–33
Set	-	2–3	-	- + 3
Repetition	-	7	-	- + 7
Days/Week	4	4	4	2 + 2
Calories Expenditure[1Min]	16.95–19.51	8.55–11.92	11.97–14.39	13.18 + 10.23
Recovery Calories Expenditure[1Min]	9.44–11.97	-	-	-
Calories Expenditure[day]	≈300.0	≈300.0	≈303.23	≈300.0
Calories Expenditure[week]	1200	1200	1214	1200

The whole-body strength exercise training was conducted for 27-33minutes a day [first week to 12 weeks] on a multi weight lifting system [AZIMUTH AZ 501D made in Iran]. The RT schedule for 12 workouts composed of 8 free weight activities [abdominal curl, bicep curl & tricep extension, dumbbell row, supine fly, heel raise, half-squat, bird-dog, hamstring curl and lateral raise]. For each muscle category, strength exercise training composed of two or three sets of 8–18 repetitions, with 90–150 s of rest between exercises and 60-120 s of rest between sets. protocol was to perform resistance exercise in the range of 55–80% of one repetition maximum. Heart rate was monitored throughout the session using a digital heart rate meter, wearing the chest belt and a wrist-strapped heart rate monitor. If the heart rate exceeded 90% HR max or reached below 70% HRmax, exercise intensity was decreased or increased, respectively. Subjects’ systolic and diastolic blood pressures were measured using standard clinical methods to ensure the health of the subjects before and after each exercise session in a rest state with the ALPK2 V-500 pressure gauge (Table 1).

patient of AT group received aerobic training monitored on bike ergometers [The Monark 894E Ergomedic Peak Bike made in Sweden] for 12 weeks on 4 days /week. The workouts consisted of warm up for 5–7 min, biking for 20–25 min and cool down for 5 min. The heart rate reserve [HRR] for every season was18-134 Equivalent 70–80% HRR that extracted from the Karvonen Formula (Table 1).

The combined AT + RT plan was structured according on the latest researches and current clinical recommendations for patients with type 2 diabetes, and continued 12 weeks with 4 days/week [2 days AT and 2 days RT]. Each workout day had one protocol. The two protocols were to carried out aerobic and resistance exercise on a bike ergometer. Resistance and aerobic exercises were performed based on the protocols mentioned above (Table 1). According to the guidelines of the present study, calories consumed in each session were approximately 300 kcal.

### Blood measurement and analyzes

Following a 10-h overnight fast and at minimum 48 h following training sessions, tests of venous blood [15 ml] were obtained. The tests obtained were centrifuged for 5 min at 3500 rpm, and processed at − 80° C until examined and the serum was isolated. The preceding procedures were employed to check the biochemical variables in the serum tests: fasting blood glucose [FBG], total cholesterol [TC], triglyceride [TG] was assessed by biochemical system Hitachi 7600 on the last day as the blood was obtained utilizing chemical alteration process. Glycosylated hemoglobin [HbA1c] has meanwhile been tested using liquid chromatography by saccharification of HA8180 machine examination. The residual sera aliquots were processed at − 80° C to examine levels of Leptin, Irisin, Sfrp5, and LGR4 utilizing commercially accessible ELISA [Zell Bío GmbH, made in Germany and Shanghai Enzyme-linked Biotechnology Co., Ltd., the brand of kits was Elabscien] kits. Actions were exclusively in accordance with the guidelines for the kit. So, Leptin was detected in the range of 0.3 to 12 μg/L, irisin was measured in the range of 6 to 280 pg. mL, LGR4 was detected in the range of 15 to 500 pg. mL, and Sfrp5 was measured in the range of 3–80 ng.L.

### Anthropometric parameters

To determine the body composition, the heights of the subjects were calculated using a 5 mm precision SECA height gage [made in Germany], and the weights of the subjects and PBFs were calculated using a body composition analyzer [InBody-720 model, South Korea] with a precision of 100 g using bioelectrical resistance system. BMI is calculated based on weight /height [kg/m2]. Once the participants avoided feeding and drinking drinks for 4 h, all measures were taken and their liver, uterus, and intestine were completely clean.

### Statistical analysis

Both measurements are displayed as mean ± SD. The Shapiro–Wilk method has been used to check the normality of data. Variance homogeneity test was performed using Levene method. After 12 weeks repeated measures analysis of variance [RM ANOVA] was performed to compare within and between subject. To investigate the relationship between variables, the Pearson correlation test was also used. The statistical analyzes is carried out using the statistical program SPSS 24.0. P values < 0.05 were considered statistically significant.

## Results

Table 1 shows the program designed for the 4 groups. Intensity and duration Exercise training programs designed based on 300 kcal energy expenditure.

Table 2 indicates the standard value of Primary physiological characteristics [age, Weight, BMI, FAT, SBP, DBP, TC, TG, LDL, HDL, FFA, FPG, 2-h PG, HbA1c, HOMA-IR] in HIIT, RT, AT and AT + AR T2DM participants did not indicated significant different in the pre-test measurements between groups.

**Table 2:** Primary physiological characteristics in HIIT, RT, AT and AT + AR T2DM participants

	HIIT	RT	AT	AT + RT
number	17	18	17	16
Age [yrs.]	52.2 ± 4.8	51.6 ± 5.0	52.8 ± 3.1	53.2 ± 3.9
Weight[kg]	102.7 ± 6.3	100.4 ± 8.1	101.7 ± 8.2	103.6 ± 6.5
BMI [kg/m2]	31.8 ± 1.3	31.6 ± 1.6	31.2 ± 1.1	32.2 ± 2.0
FAT [%]	36.4 ± 4.5	34.2 ± 3.9	35.5 ± 3.1	35.2 ± 4.2
SBP [mm Hg]	128.4 ± 13.2	125.9 ± 11.7	123.9 ± 10.7	124.8 ± 9.3
DBP [mm Hg]	80.2 ± 9.8	79.8 ± 7.1	82.6 ± 10.2	80.3 ± 9.5
TG [mmol/liter]	1.99 ± 0.86	2.01 ± 1.11	2.12 ± 0.72	2.03 ± 0.83
TC [mmol/liter]	4.80 ± 1.02	4.63 ± 1.15	5.00 ± 0.99	4.85 ± 1.02
HDL-C [mmol/liter]	1.30 ± 0.73	1.45 ± 0.89	1.42 ± 1.03	1.35 ± 0.97
LDL-C [mmol/liter]	2.60 ± 1.15	2.68 ± 0.93	2.55 ± 1.43	2.19 ± 1.22
FFA [umol/liter]	0.66 ± 0.32	0.70 ± 0.29	0.68 ± 0.85	0.69 ± 0.61
FPG [mmol/liter]	9.92 ± 3.04	9.10 ± 4.16	10.26 ± 4.67	9.55 ± 5.63
2-h PG [mmol/liter]	16.95 ± 2.51	17.02 ± 3.72	17.26 ± 2.03	17.11 ± 3.43
HbA1c [%]	8.12 ± 1.55	8.88 ± 1.83	9.03 ± 2.08	8.17 ± 2.15
HOMA-IR	5.25 ± 1.93	5.48 ± 1.35	4.66 ± 2.34	5.04 ± 2.07

**Fig. 2:**
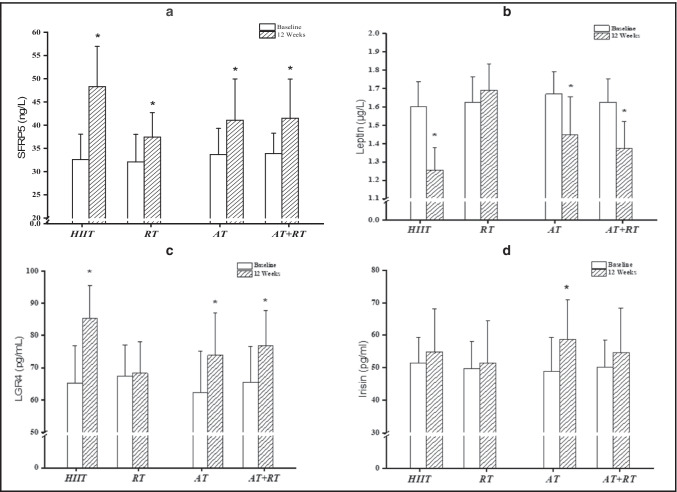
Adipocytokines profile baseline and after 12 weeks. high intensity interval training **[HIIT]**, Resistance Training **[RT]**, Aerobic Training **[AT]** and Resistance Training + Aerobic Training **[AT + RT]**. SFRP5(**a**), Leptin(**b**), LGR4(**c**) & Irisin (**d**). * Significant difference 12 weeks compared with baseline training [P < 0.05]. data are demonstrated as mean ± SD

About Fig. 2a, there is significant difference for the concentration of SFRP5 resting level between the training groups [HIIT, AT, RT and RT + AT]. A 2 × 4 repeated measures ANOVA was used to investigate the data obtained from the effects of exercise on SFRP5. The results of the Shapiro–Wilk test showed that the blood SFRP5 level has a normal distribution for all training groups in pre-test and post-test sessions [p > 0.05]. According to results, the Levin test shows that there is homogeneity of variance between the scores of the training groups in the pre-test and post-test [p > 0.05]. the results of the repeated measures ANOVA show that the effect of the test is significant, between groups [F [3,64] = 0.027], within groups [F [1,64] = 0.001] Test * Group [F [3,64] = 0.003] also was significant. Also, Fig. 2, the findings revealed that the concentration of SFRP5 resting in the study groups increased significantly after 12 weeks exercise training intervention [from 32.59 ± 5.46 to 48.28 ± 8.68 in HIIT group; from 32.08 ± 5.94 to 37.43 ± 5.25 in RT group; from 33.67 ± 5.65 to 41.05 ± 8.88 in AT group; from 33.86 ± 4.41 in AT + RT group].

About Fig. 2b, there is significant difference for the concentration of leptin resting level between the training groups [HIIT, AT, RT and RT + AT]. A 2 × 4 repeated measures ANOVA was used to investigate the data obtained from the effects of exercise on leptin. The results of the Shapiro–Wilk test showed that the blood leptin level has a normal distribution for all training groups in pre-test and post-test sessions [p > 0.05]. According to results, the Levin test shows that there is homogeneity of variance between the scores of the training groups in the pre-test and post-test [p > 0.05].

The results of the repeated measures ANOVA show that the effect of the test is significant, between groups [F [3,64] = 0.001], within groups [F [1,64] = 0.001] Test * Group [F [3,64] = 0.001] also was significant.

Figure 2b also shows a significant decrease in the leptin following 12 weeks exercise training program in three groups [Except in RT group]. [in HIIT, AT and AT + RT from 1.60 ± 0.13 to 1.25 ± 0.12, from 1.67 ± 0.12 to 1.44 ± 0.20 and from 1.62 ± 0.12 to 1.34 ± 0.14, respectively] [P = 0.001, P = 0.001 and P = 0.001].

About Fig. 2c, there is significant difference for the concentration of LGR4 resting level between the training groups [HIIT, AT, RT and RT + AT]. A 2 × 4 repeated measures ANOVA was used to investigate the data obtained from the effects of exercise on LGR4. The results of the Shapiro–Wilk test showed that the blood LGR4 level has a normal distribution for all training groups in pre-test and post-test sessions [p > 0.05]. According to results, the Levin test shows that there is homogeneity of variance between the scores of the training groups in the pre-test and post-test [p > 0.05].

The results of the repeated measures ANOVA show that the effect of the test is significant, between groups [F [3,64] = 0.048], within groups [F [1,64] = 0.001] Test * Group [F [3,64] = 0.002] also was significant.

The findings of the repeated measure test in Fig. 2c indicates that HIIT, AT and AT + RT groups increased serum concentrations of LGR4 after 12 weeks interventions as opposed to baseline test [in HIIT, AT and AT + RT from 65.09 ± 11.84 to 85.21 ± 10.19, from 62.26 ± 12.92 to 73.83 ± 13.30 and from 65.49 ± 11.05 to 76.92 ± 10.77, respectively] [P = 0.001, P = 0.001 and P = 0.001].

About Fig. 2d there is no significant difference for the concentration of irisin resting level between the training groups [HIIT, AT, RT and RT + AT]. A 2 × 4 repeated measures ANOVA was used to investigate the data obtained from the effects of exercise on irisin. The results of the Shapiro–Wilk test showed that the blood irisin level has a normal distribution for all training groups in pre-test and post-test sessions [p > 0.05]. According to results, the Levin test shows that there is homogeneity of variance between the scores of the training groups in the pre-test and post-test [p > 0.05]. The results of the repeated measures ANOVA show that the effect of the test is significant. Figure 2d also shows About irisin, findings have also demonstrated that after 12 weeks interventions as compared with basic conditions, there is an increased significantly in AT groups [F [3,64] = 0.018] only than other groups.

**Fig. 3:**
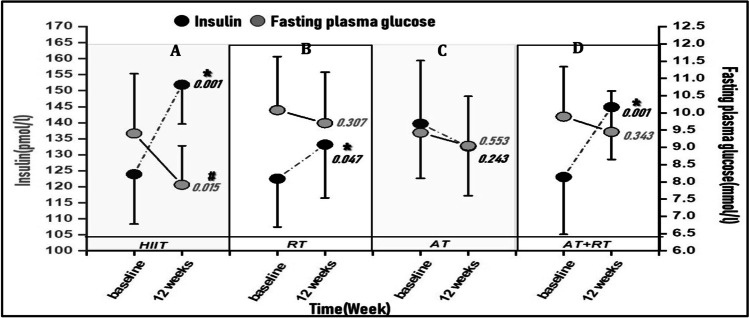
Insulin vs Glucose profile baseline and after 12 weeks. high intensity interval training **[HIIT]**, Resistance Training **[RT]**, Aerobic Training **[AT]** and Resistance Training + Aerobic Training **[AT + RT]**. HIIT(**a**), RT(**b**), AT(**c**) & AT + RT (**d**). * Significant difference Fasting plasma glucose12 weeks compared with baseline training; **#** Significant difference Insulin 12 weeks compared with baseline training [P < 0.05]. data are demonstrated as mean ± SD

Figure 3a-d The results of the repeated measures ANOVA indicates the Insulin vs Glucose for the two conditions at before, after 12 weeks. After 12 weeks, A Significant increased after the HIIT [P = 0.001], AT [P = 0.047] and AT + RT [P = 0.001] in comparison to the baseline. In contrast, Fig. 3a-d shows only Significant decreased after the HIIT [P = 0.015], in comparison to the baseline.

Table 3 shows that After the 12 weeks exercise training programs, all group showed positive changes in weight[p = 0.001, p = 0.001, p = 0.003 and p = 0.001 for HIIT, RT, AT, AT + RT respectively], BMI[p = 0.001, p = 0.001, p = 0.001 and p = 0.001 for HIIT, RT, AT, AT + RT respectively], SBP[p = 0.002, p = 0.005, p = 0.001 and p = 0.005 for HIIT, RT, AT, AT + RT respectively], DBP[p = 0.001, p = 0.001, p = 0.001 and p = 0.001 for HIIT, RT, AT, AT + RT respectively] and 2hPG[p = 0.001, p = 0.024, p = 0.001 and p = 0.001 for HIIT, RT, AT, AT + RT respectively], while NO group showed positive changes in LDL. Table 3 Also shows that HDL only decreased significantly in HIIT than other groups [p = 0.002]. About FFA in Table 3, presented that TG and TC decreased significantly in HIIT and AT + RT. After intervention period HbA1c decreased significantly in HIIT, AT and AT + RT [p = 0.002, p = 0.026, p = 0.026 respectively]. Tables 4 and 5 show the relationship between the variables.

**Table 3:** Anthropometrics, physiologic & glycemic and lipidemic responses to HIIT, RT, AT and AT + RT at baseline vs 12 weeks exercise training

	Variable	weight	BMI	SBP	DBP	LDL	HDL	TG	TC	FFA	HbA1c	2hPG
***HIIT***	***baseline***	101.70 ± 5.68	31.83 ± 1.19	125.12 ± 6.25	84.02 ± 5.76	1.35 ± 0.48	2.52 ± 0.62	1.94 ± 0.58	4.94 ± 0.57	9.96 ± 2.42	8.22 ± 0.94	17.01 ± 1.03
	***12 weeks***	86.93 ± 3.55	28.70 ± 1.60	120.33 ± 4.50	78.34 ± 4.54	1.06 ± 0.32	3.15 ± 0.67	1.37 ± 0.27	4.04 ± 0.50	9.13 ± 1.83	7.20 ± 0.82	14.06 ± 1.50
	***sig***	***0.001***	***0.001***	***0.002***	***0.001***	***0.056***	***0.002***	***0.002***	***0.001***	***0.001***	***0.002***	***0.001***
***RT***	***baseline***	102.19 ± 3.98	31.92 ± 30.51	126.38 ± 5.15	84.06 ± 6.44	1.47 ± 0.43	2.82 ± 0.63	1.92 ± 0.62	4.77 ± 0.63	10.43 ± 2.38	8.12 ± 0.92	16.48 ± 1.06
	***12 weeks***	94.91 ± 3.98	30.51 ± 1.06	120.91 ± 5.74	78.81 ± 6.17	1.21 ± 0.46	2.76 ± 0.63	1.73 ± 0.47	4.54 ± 0.44	9.94 ± 1.79	7.72 ± 0.77	15.69 ± 1.04
	***sig***	***0.001***	**0.001**	**0.005**	**0.001**	**0.119**	**0.801**	**0.398**	**0.290**	**0.290**	**0.062**	**0.024**
***AT***	***baseline***	98.49 ± 5.17	32.20 ± 1.39	127.10 ± 5.44	82.60 ± 5.46	1.38 ± 0.50	2.53 ± 0.82	20.07 ± 0.66	4.51 ± 0.64	10.44 ± 2.31	8.14 ± 0.97	17.10 ± 1.17
	***12 weeks***	92.56 ± 3.90	29.99 ± 1.40	117.89 ± 5.65	74.91 ± 6.98	1.13 ± 0.39	2.81 ± 0.65	1.83 ± 0.38	4.50 ± 0.41	9.27 ± 2.20	7.25 ± 0.82	15.34 ± 0.62
	***sig***	***0.003***	***0.001***	***0.001***	***0.001***	***0.072***	***0.307***	***0.238***	***0.927***	***0.927***	***0.026***	***0.001***
***AT* + *RT***	***baseline***	101.97 ± 5.37	31.51 ± 1.21	125.96 ± 6.54	83.41 ± 7.01	1.30 ± 0.46	2.41 ± 0.67	1.98 ± 0.61	4.93 ± 0.58	10.55 ± 2.47	8.06 ± 0.79	17.27 ± 1.03
	***12 weeks***	92.40 ± 3.05	29.82 ± 1.32	118.62 ± 4.43	75.84 ± 4.50	1.19 ± 0.43	2.89 ± 0.64	1.59 ± 0.28	4.46 ± 0.46	9.59 ± 1.64	7.40 ± 0.95	14.60 ± 0.98
	***sig***	***0.001***	***0.001***	***0.005***	***0.001***	***0.344***	***0.099***	***0.034***	***0.009***	***0.009***	***0.026***	***0.001***

*Significant difference [*P* < 0.05]. Data are demonstrated as mean ± SD

**Table 4:** Correlations of SFRP5, Leptin, LGR4& Irisin and anthropometrics and physiologies parameters in 4 groups

Variables		weight	BMI	SBP	DBP	LDL	HDL	TG	TC	FFA	HbA1c	2hPG	
HIIT	SFRP5	r	-0.559*	-0.893*	-0.758*	0.258	-0.502*	0.555*	-0.715*	-0.490	-0.443	-0.912*	-0.249
		P-value	0.020	0.001	0.001	0.320	0.039	0.077	0.001	0.088	0.135	0.001	0.329
	Leptin	r	0.857*	0.912*	0.437	0.366	0.736*	0.462	0.145	0.273	0.552*	0.882*	0.601*
		P-value	0.001	0.001	0.141	0.212	0.001	0.116	0.433	0.305	0.027	0.001	0.001
	LGR4	r	-0.699*	-0.843*	-0.281	-0.210	-0.499	-0.251	-0.342	-0.353	-0.421	-0.568*	-0.843*
		P-value	0.001	0.001	0.297	0.368	0.079	0.327	0.236	0.225	0.157	0.011	0.001
	Irisin	r	-0.720*	-0.543*	-0.320	-0.254	-0.488	-0.520	-0.482	-0.412	-0.380	-0.849*	-0.683*
		P-value	0.001	0.036	0.258	0.324	0.090	0.059	0.096	0.166	0.198	0.001	0.001
RT	SFRP5	r	-0.755*	-0.710*	-0.689*	-0.882*	-0.407	-0.125	-0.222	-0.310	-0.355	-0.394	-0.620*
		P-value	0.001	0.001	0.001	0.001	0.171	0.453	0.356	0.268	0.223	0.184	0.001
	Leptin	r	0.482*	0.775*	0.122	0.227	0.405	0.367	0.219	0.165	0.382	0.790*	0.825*
		P-value	0.096	0.001	0.456	0.351	0.173	0.211	0.359	0.413	0.196	0.001	0.001
	LGR4	r	-0.699*	-0.820*	-0.190	-0.249	-0.440	-0.297	-0.183	-0.264	-0.324	-0.809*	-0.643*
		P-value	0.001	0.001	0.388	0.329	0.138	0.281	0.395	0.314	0.254	0.001	0.001
	Irisin	r	-0.438	-0.227	-0.145	-0.358	-0182	-0.352	-0.297	-0.417	-0.120	-0.344	-0.335
		P-value	0.140	0.351	0.433	0.220	0.396	0.226	0.281	0.161	0.457	0.234	0.243
AT	SFRP5	r	-0.620*	-0.588*	-0.571*	-0.549*	-0.305	-0.246	-0.288	-0.192	-0.194	-0.719*	-0.932*
		P-value	0.001	0.006	0.016	0.036	0.155	0.290	0.264	0.586	0.601	0.001	0.001
	Leptin	r	0.551*	0.711*	0.274	0.184	0.103	0.319	0.193	0.246	0.531*	0.752*	0.695*
		P-value	0.028	0.001	0.304	0.394	0.475	0.259	0.385	0.332	0.048	0.001	0.001
	LGR4	r	-0.619*	-0.876*	-0.131	-0.202	-0.351	-0.401	-0.219	-0.237	-0.427	-0.686*	-0.888*
		P-value	0.001	0.001	0.447	0.376	0.227	0.177	0.359	0.341	0.151	0.001	0.001
	Irisin	r	-0.549	-0.722	-0.257	-0.457	-0.234	-0.124	-0.143	-0.333	-0.255	-0.589	-0.667
		P-value	0.030	0.001	0.321	0.121	0.344	0.454	0.435	0.245	0.323	0.001	0.001
AT + RT	SFRP5	r	-0.694*	-0.600*	-0.933*	-0.266	-0.289	-0.790*	-0.825*	-0.407	-0.474	-0.876*	-0.562*
		P-value	0.001	0.001	0.001	0.312	0.289	0.001	0.001	0.171	0.104	0.001	0.017
	Leptin	r	0.530*	0.890*	0.191	0.257	0.225	0.412	0.353	0.483	0.128	0.822*	0.539*
		P-value	0.049	0.001	0.387	0.321	0.353	0.166	0.225	0.095	0.450	0.001	0.040
	LGR4	r	-0.755*	-0.618*	-0.334	-0.145	-0.312	0.099	0.198	-0.246	-0.279	-0.672*	-0.606*
		P-value	0.001	0.001	0.244	0.433	0.266	0.479	0.380	0.332	0.303	0.001	0.001
	Irisin	r	-0.301	-0.380	-0.223	-0.255	-0.254	-0.482	-0.138	-0.173	-0.386	-0.488	-0.465
		P-value	0.277	0.198	0.355	0.323	0.324	0.096	0.440	0.405	0.192	0.090	0.113

**Table 5:** Correlations of SFRP5, Leptin, LGR4& Irisin with toghater in 4 groups

Variables		HIIT			RT			AT			AT + RT		
		Leptin	LGR4	Irisin	Leptin	LGR4	Irisin	Leptin	LGR4	Irisin	Leptin	LGR4	Irisin
SFRP5	r	-0.724*	0.802*	0.125	0.458	0.167	0.375	-0.567*	0.620*	0.680*	-0.625*	0.665*	0.565*
	P-value	0.001	0.001	0.453	0.120	0.411	0.203	0.012	0.001	0.001	0.001	0.001	0.014
Leptin	r		-0.692*	-0.200		0.050	0.195		-0.499	-0.560*		-0.530*	-0.360
	P-value		0.001	0.378		0.528	0.383		0.079	0.019		0.049	0.218
LGR4	r			+ 0.155			0.076			0.610*			0.438
	P-value			0.423			0.502			0.001			0.140

## Discussion

It is the first research, to our information, to explore the impact of four separate forms of exercise training with the same energy expenditure on circulating irisin and its correlation with anthropometrics and physiological variables in obese with T2D men. Thus, the target of this research is to examine variations in adipocytokines [SFRP5, leptin, LGR4 and irisin] after 12 weeks of 4 types exercise training [high intensity interval training, resistance training, aerobic training and aerobic + resistance training] and as well as effects exercise Training Induced Calories Expenditure on Type 2 Diabetes Related Cardio Metabolic Physiological Parameters and adipocytokines in T2D patients. This research revealed that 12 weeks of training programs with equal energy expenditure in T2D significantly increased levels of concentration of SFRP5[at 4 groups], LGR4[expect in RT group] and irisin [only at AT group] and significantly decreased levels of concentration of leptin [expect in RT group]. Also, Type 2 Diabetes Related Cardio Metabolic Physiological Parameters [weight, BMI, SBP, DBP, HDL, TG, TC, FFA, HbA1c and 2hPG] significantly improvement after 12 weeks Interventions. After Intervention period insulin significantly increased in HIIT, RT and AT + RT groups. While, fasting plasma glucose significantly decreased in HIIT group only.

Irisin may be considered as a powerful marker for reducing metabolic predisposing factors in IR and T2D. this adipocytokines result in the browning of white adipose tissue, facilitating glucose uptake in the many tissues[such as heart, skeletal muscle and liver], and promoting pancreatic β cell activity by increasing hepatic glucose and lipid metabolism [25]. This study demonstrated that HIIT, RT, AT and AT + RT in T2D men had impact on circulating irisin [HIIT: %6.6; RT: %3.5; AT: %20.4 and AT + RT: %9.2]. nevertheless, after 12 weeks of exercise training, AT group showed higher circulating irisin amount relative to other categories. However, the present study has showed no significantly different between 4 groups [F = 0917, P = 0.438]. Also, date has indicated no relationship between bloodstream irisin concentrations and anthropometrics and physiological variables. Our results were compatible with the research published by Huh et al. that documented which improvements in bloodstream irisin concentrations were not significantly different after high-intensity intermittent exercise [HIIE], continuous moderate exercise [CME] and resistance exercise [RE] [26].

Furthermore, in a T2D condition, adaptation responses to exercise training can be stronger relative to regular metabolic condition. Additionally, HIIT and AT, RT and AT + RT have had the same effect on improvements in circulating irisin concentrations, However, in the aerobic training group [%20.4], this effect was more than other groups. This may be the product of the metabolic disorder. Our results differ from the research performed by Kartinah et al. [2018] demonstrated that the impact of HIIT on adipose irisin concentrations was seen to be significantly higher compared to continuous moderate intensity training [CMIT] in unhealthy metabolic diseases [18]. Also, Shabani et al. [2018] were observed that the concentrations of serum irisin were significantly decreased by the [aerobic + resistance] exercise training [CET] group compared with resistance exercise training [RET], and aerobic exercise training [AET]. In fact, they revealed RET and AET in formerly untrained young women had no influence on serum irisin [27]. The difference between the Shabani study and the present study may be due to the energy consumed. Because in the present study, energy consumption was equal to the four types of exercise training. Given that energy expenditure was equal in the four training groups. Therefore, it seems that other mechanisms besides energy expenditure are effective in increasing irisin levels. However, studies have shown that irisin is associated with some anthropometric indices such as BMI and weight [18, 27, 28]. But in the present study, it was shown that after 12 weeks of intervention, BMI, weight, HbA1c and 2 h-PG was only associated with irisin in the HIIT group. Although HIIT may be associated with BMI and weight as well as glycemic indexes due to high intensity [18], but, it is not observed in aerobic and resistance training and combination due to low intensity and type of exercise than RT, AT and AT + RT. The mechanisms of exercise training in influencing irisin changes are still unknown. However, Irisin’s function in metabolism is mirrored in a rise in energy consumption and homeostasis to glucose. hence it could be reasonable that irisin and FNDC5 are typically distinguished by enhanced oxidative capacity and mitochondrial capacities in response to aerobic exercise than resistance training and AT + RT. Nevertheless, there was an increase in other groups, it is also likely that HIIT, RT and AT + RT anabolic effects [muscle development] are correlated with enhanced FNDC5 expression, because in a research investigator revealed irisin was mainly correlated with muscle mass.

Other authors have noted that High concentration of serum irisin in obese subjects and low concentration of serum irisin in T2DM patients might indicate its defensive function toward insulin resistance. Saleh et al. [2014] The average amount of serum irisin levels in obese healthy persons was the maximum level in contrast with normal and overweight healthy persons [29]. But, in present study, these changes may be different after 12 weeks of exercise. because, these changes were not observed after the interventions between irisin and AT and AT + RT.

Irisin promotes skeletal muscle consumption of glucose, increases hepatic glucose and lipid metabolism, and has a beneficial impact on hyperlipidemia and hyperglycemia induced by obesity and metabolic syndrome [30]. And hence functions as the hormone sensitizing insulin [25]. Irisin is thought to impact organs and tissues engaged in type 2 diabetes, such as liver and pancreas, by rising IR, but the mechanisms by which it modulates the pancreatic islet activity are still unclear. Irisin is proposed to enhance hepatic metabolism by decreasing endoplasmic reticulum stress [ER stress], and to lead to the survival and functioning of β-islet cell mass [31].

The findings of this study indicate positive alter of irisin during the study can be explained by exercise training induced FNDC5 protein expression. However, the amount of FNDC5 protein expression maybe different in exercise training with the expenditure of energy consumption. And aerobic exercise is likely to express greater amounts of this gene than other groups.

Other findings of our experiment indicate that HIIT [%-21.7], AT [%-13.3] and AT + RT [%-15.4] significantly decreased of leptin concentration after 12 weeks intervention. Also, results showed that HIIT has positive correlation with weight, BMI, LDL, FFA, Hba1C and 2hPG; and AT has positive correlation with weight, BMI, FFA, HbA1c and 2hPG and RT and AT + RT have positive correlation with weight, BMI,2 h-PG, HbA1c. Racil et al. [2016] showed a Significant decrease in blood leptin in HIIT and MIIT, respectively. Blood glucose, insulin level and the homeostasis model assessment index for insulin decreased in both training groups. In the post-intervention period, blood leptin was strongly associated with %BF and VO2max in the HIIT and MIIT groups, respectively [9]. based on the literature, leptin functions on the central nervous system through decreasing appetite and by increasing energy consumption [32–34]. Aktas et al. [2019] When PCOS patients by completed HIIT Compared with Medium Intensity Continuous Training [MICT] for 12 weeks, they observed that the concentrations of leptin did not increase. In turn, the serum insulin concentrations, TG, total cholesterol, LDL-C reduced while the HDL-C concentrations raised [33],Which is in line with the findings of this study. Avazpor et al. [2016] also confirmed the findings [35]. Although it is noted that training intensities [high and moderate and even low] improved aerobic ability and some of anthropometrics and physiological variables, improved concentrations of leptin in the bloodstream [9, 36]. However, it seems that in this study, increasing the intensity of exercise training as well as the type of exercise intervention, leptin levels showed a significant decrease. Therefore, one may conclude that perhaps the findings that address the clinical consequences of leptin induced exercise training are already quite inconsistent. Therefore, based on the findings of the present study, If the energy expenditure of exercise is equal, HIIT and AT + RT will significantly reduce the concentration of leptin. Hence, the effectiveness of HIIT as well as AT + RT was more effective than aerobic and resistance training alone.

In this study, it has been demonstrated that the highest percentage of changes in the SFRP5 belonged to the HIIT training group, like leptin. However, in all groups [HIIT: %48.1; RT: %16.7; AT: %21.9 and AT + RT: %22.5], this anti-inflammatory marker significantly increased. SFRP5 is a currently identified adipocytokine related to obesity-related insulin resistance and type 2 diabetes and reduced inflammation [37]. in extracellular adipose tissue space, SFRP5 attaches WNT5A and it may serve as an effective trigger for pro-inflammation. SFRP5 coupling to WNT5A is suspected to mitigate Wnt signaling [38]. The expression of Sfrp5 in obese leptin deficient [ob / ob] mice was reduced [39]. Evidence on SFRP5 and cardiometabolic disorders are ambiguous in populations. Cross trials for those with and/or without type 2 diabetes reported that SFRP5 was direct and indirect and no association with glycemic and cardiometabolic variables [40, 41]. Based on our knowledge, the present study appears to be the first research to examine four types of exercise training interventions on SFRP5marker. Therefore, it can be concluded that SFRP is more sensitive to the type and intensity of exercise, compared to leptin and irisin. If the exercise training programs are performed at the same energy expenditure, higher intensity training is likely to have a greater impact on anti-inflammatory markers than pre-inflammatory markers. However, this theory may be true in obese type 2 diabetic patients and not in healthy individuals. In agreement with our results, Fayaz et al.[2019] showed that, coupled with aqueous cinnamon extract supplementation for 12 weeks, high-endurance training more effectively ameliorated insulin resistance and metabolic disorders by suppression of noncanonical WNT signaling in ovariectomized rats [42]. Also et al. found that both obese mice and diabetic rats fed high-fat diets with SFRP5 expression was downregulated [39]. Which was in line with the results of this study. The outcomes of this research are compatible with recent studies and have established that HIIT in type 2 diabetes results in more improvements in body mass, BMI, 2 h-PG, TC, TG, HDL, LDL, insulin secretion, HbA1c and HOMA-IR Compared to other groups [9, 43]. Therefore, it can be concluded that the change in the SFRP5 as an anti-inflammatory marker has probably been able to affect the cardiometabolic and glycemic indexes, and/or perhaps the changes in these indicators have been able to increase the SFRP5. Overall, HIIT appears to reduce inflammation in type 2 diabetics by more effective on many those factors.

The results of this study are the first time to examine the effect of exercise on LGR4 in type 2 diabetic patients. However, based on our knowledge, so far, no research has examined the effect of exercise on this variable in either patients or healthy individuals. As it turns out, most of the changes are specific to the HIIT group [%30.9], and the changes in the AT and AT + RT are almost close to each other [%18.6 VS %17.5], and the lowest changes are for the RT [%1.3] group. Authorities Also discovered that LGR4 is severe in islets and expressed that LGR4 mediate the pancreatic function of R-spondins [44, 45]. The concentrations of LGR4 in T2D that performed HIIT is highest relative to those performed, AT + RT and even AT and RT only. Further evidence showed that weight, BMI, HbA1c, and 2 h-PG were adversely linked to plasma concentrations of LGR4 in 4 groups. Whereas the findings indicated that SFRP5, leptin and LGR4 positive changes with elevated intensity of exercise training [HIIT], while, Irisin changed with AT. Overall, is thought that exercise training with similar energy expenditure result in improve pro-inflammatory and anti-inflammatory cytokines. But there is speculation as to why exercise training at the same energy expenditure causes different changes in pro-inflammatory and anti-inflammatory markers. Therefore, more studies are needed in the future to clarify the mechanisms that affect these changes.

In this study, HIIT group indicated a strong negative association between leptin and SFRP5 and LGR4 was observed. In this group, the data also showed a positive association between leptin and SFRP and LGR4. In the AT group, we found that leptin had a strong negative relationship with SFRP5 and irisin. Irisin also showed a positive and significant correlation with SFRP5 and LGR4. However, there is a positive relationship between LGR4 and SFRP5. The findings also found a negative correlation between leptin and SFRP5 and LGR4. SFRP5 showed a positive relationship with LGR4 and Irisin. These variables involve a variety of mechanisms, including: Irisin has been characterizes as a [PGC1α]-dependent adipocytokine, the same as Leptin, which takes action by AMPK signaling pathway in controlling blood glucose in normal and T2D [25]. Irisin can induce body mass loss by growing energy consumption and induces elevated uncoupling protein 1 [UCP1] and e expression of the transmembrane protein fibronectin type III domain-containing [26]. In addition, circulating irisin is positively associated with lean mass while fat mass is inversely associated. Consequently, in humans, irisin concentrations were positively associated with variables of adiposity such as body mass, % fat, BMI, WHR, and IR and HOMA-IR [16]. SFRP5 is recently recognized as an anti-inflammatory adipocytokine and Compared to other tissues is strongly expressed in white adipose tissue [WAT] [37]. It has been stated that adipogenesis is inhibited by the canonical Wnt family, whereas non-canonical Wnt5a strengthens the signaling pathway to the inflammation. When caused by obesity, SFRP5 attaches to and prevents Wnt5a [16]. Leptin is a traditional pro-inflammatory adipokine and is mainly released into the circulation by adipocytes. This also facilitates the formation of reactive oxygen species [ROS] and induces monocyte cell proliferation and migratory actions [33]. Leptin stimulates the generation of CC-chemokine ligands on macrophages by triggering the JAK2/STAT3 signaling pathway. Moreover, in patients with type II diabetes, the circulating leptin concentrations tend to decrease following exercise [13].

Given the link between inflammatory and pre-inflammatory markers in the present study, it appears that these changes in exercise training with equal energy expenditure will improve glycemic and lipid indexes. Overall, these changes improve the condition of type 2 diabetics. The results of these changes can be seen in the improvement of insulin secretion and fasting glucose.

### Clinical implication

The findings of the present study showed that exercising at the same cost of energy causes different changes in pre-inflammatory and inflammatory indicators.

High-intensity interval training at the same energy cost as other exercise training will further improve type 2 diabetic patients.

The effectiveness of HIIT on glycemic and lipidemic indicators is higher than other aerobic and resistance training and even resistance and aerobic combination.

## Footnotes

1 Total Cholesterol.

2 Triglycerides.

3 reactive oxygen species.

4 Continuous Moderate-Intensity Training.

5 Leucine-rich repeat-containing G protein-coupled receptors-4.
